# Induced Pluripotent Stem Cell-Derived Dendritic Cells Provide a Reliable In Vitro Platform for Functional Screening of Immunoregulatory Probiotics

**DOI:** 10.3390/ijms27010303

**Published:** 2025-12-27

**Authors:** Yin-Ling Chiang, Men-Yee Chiew, Sheng-Jye Lim, Ding-Li Chou, Huai-En Lu, Ching-Ping Tseng

**Affiliations:** 1 Industrial Development Graduate Program of College of Engineering Bioscience, National Yang Ming Chiao Tung University, Hsinchu 300, Taiwan; candyi8113@gmail.com; 2 Center for Regenerative Medicine and Cellular Therapy, National Yang Ming Chiao Tung University, Hsinchu 300, Taiwan; chiewmy@nycu.edu.tw (M.-Y.C.); shengjye2022@nycu.edu.tw (S.-J.L.); 3 Department of Biological Science and Technology, College of Engineering Bioscience, National Yang Ming Chiao Tung University, Hsinchu 300, Taiwan; gs12099@yahoo.com.tw

**Keywords:** induced pluripotent stem cell, dendritic cell, probiotic, co-culture, CD4^+^ T cell

## Abstract

The immunoregulatory effects of probiotics have been widely studied, particularly in maintaining immune balance. Conventional in vitro functional screening of probiotics relies on fresh donor-derived primary immune cells, which often exhibit significant inter-individual and temporal variability, limiting reproducibility and interpretation. As an alternative, human-induced pluripotent stem cell (iPSC)-derived dendritic cells were co-cultured with five probiotic strains in the current study to evaluate their immunomodulatory interactions. To assess whether cytokines produced by probiotic-stimulated dendritic cells can influence T cell differentiation, human CD4^+^ T cells were exposed to the conditioned medium derived from co-cultures. Enzyme-linked immunosorbent assay results demonstrated that iPSC-derived dendritic cells secreted cytokines at distinct concentrations in response to different probiotic strains, suggesting that these cells can distinguish between different microbial stimuli, and supporting their use in functional probiotic screening. Among the five strains tested, *Lactiplantibacillus plantarum* LPA-56, *Limosilactobacillus reuteri* RU-23, and *Lactobacillus fermentum* Fem-99 induced cytokine production levels that promoted the differentiation of the human CD4^+^ T cells into regulatory T cells. These findings demonstrate that iPSC-derived dendritic cells have immunomodulatory potential, are reliable for in vitro screening of probiotics, and offer a promising strategy for selecting potent immunoregulatory probiotic candidates.

## 1. Introduction

Recent studies have demonstrated that the diversity of the gut microbiota, as well as fluctuations in specific microbial populations, is closely associated with human health [1]. With the implementation of the Human Microbiome Project and advances in metagenomic technologies, the link between gut microbiota dysbiosis caused by different dietary intakes and various diseases has been well-established [2], including irritable bowel syndrome and inflammatory bowel disease [3,4], as well as metabolic and cardiovascular conditions such as obesity, diabetes, and heart disease [5,6,7]. Emerging evidence has also linked gut microbiota dysbiosis to neurodevelopmental and neurodegenerative disorders, including autism, Alzheimer’s disease, and even cancer [8,9,10]. Therefore, modulation of the gut microbiota has been associated with reduced disease incidence and may offer therapeutic benefits. Probiotics have been extensively investigated not only for their ability to influence microbial community structure, but also for their capacity to exert direct physiological effects through the production of bioactive metabolites. These metabolites include short-chain fatty acids that support intestinal barrier integrity and immune homeostasis [11] as well as neuroactive compounds such as γ-aminobutyric acid (GABA), which have been implicated in metabolic regulation and mental health [12]. In this context, lactic acid bacteria are frequently investigated for their potential probiotic properties, although only strains with demonstrated health benefits are formally classified as probiotics [13]. Additionally, probiotics influence intestinal epithelial and immune cells [14,15] by enhancing intestinal barrier integrity, reducing inflammation, and modulating immune responses [14,16], with dendritic cells playing a pivotal role in these processes [15]. Probiotic–immune cell co-culture is widely used to assess the immunomodulatory properties of specific strains. This technique enables efficient in vitro screening of probiotics with functional immunomodulatory properties [17,18]. Importantly, many of these immunological benefits arise from strain-specific functional properties, highlighting the need for reliable and standardized platforms to evaluate probiotic immunomodulatory functions in a human-relevant context.

Among the probiotic strains that have been assessed to date, *Lactobacillus rhamnosus* GG is one of the most extensively studied and clinically applied probiotics [19]. It has been shown to promote the differentiation of naïve T cells into Th1 cells [20,21], and the findings of recent studies have provided further evidence of its potential efficacy in enhancing the expansion of regulatory T (Treg) cells [22]. Given its well-characterized immunomodulatory properties, *L. rhamnosus* GG is often used as a reference strain in studies investigating the interactions between probiotic and immune cells.

Dendritic cells used in current research are primarily obtained from mouse spleens or generated by differentiating peripheral blood mononuclear cells (PBMCs) [23,24]. However, primary dendritic cells are scarce, and multiple rounds of cell collection are often required to obtain sufficient quantities. Moreover, PBMCs obtained from different donors exhibit considerable heterogeneity, resulting in variability in the functional performance of differentiated dendritic cells [25,26], which poses challenges for consistent evaluation of probiotics in dendritic cell-based in vitro co-culture systems.

In 2006, Takahashi and Yamanaka successfully established a technique for generating induced pluripotent stem cells (iPSCs) by expressing the transcription factors OCT4, KLF4, SOX2, and c-MYC in somatic cells [27]. Unlike primary cells, which have limited proliferative capacity due to replicative senescence, iPSCs can expand indefinitely in culture and are a valuable source of various cell types for experimental studies. iPSC-derived dendritic cells have been utilized in disease modeling [28], mechanistic studies [29,30], cancer vaccine research [31], and even drug screening [32]. Despite these diverse applications, their use in functional probiotic screening has not yet been explored.

In this study, we used iPSC-derived dendritic cells to screen lactic acid bacteria for their immunomodulatory effects based on co-culture assays. The immunomodulatory potential of conditioned medium derived from probiotic-activated dendritic cells was further validated using human CD4^+^ T cells. By adopting this approach, we aimed to address the scarcity and donor-to-donor variability of immune cells that often hinder reproducibility in probiotic research [26]. To the best of our knowledge, this study represents the first application of iPSC-derived dendritic cells for the purpose of in vitro functional probiotic screening. We envisage that this type of platform may contribute to standardizing functional assays and accelerate the discovery of next-generation probiotics with therapeutic relevance for the treatment of immune-mediated disorders.

## 2. Results

### 2.1. Differentiation of Human iPSCs into Hematopoietic Stem Cells

**Figure 1:**
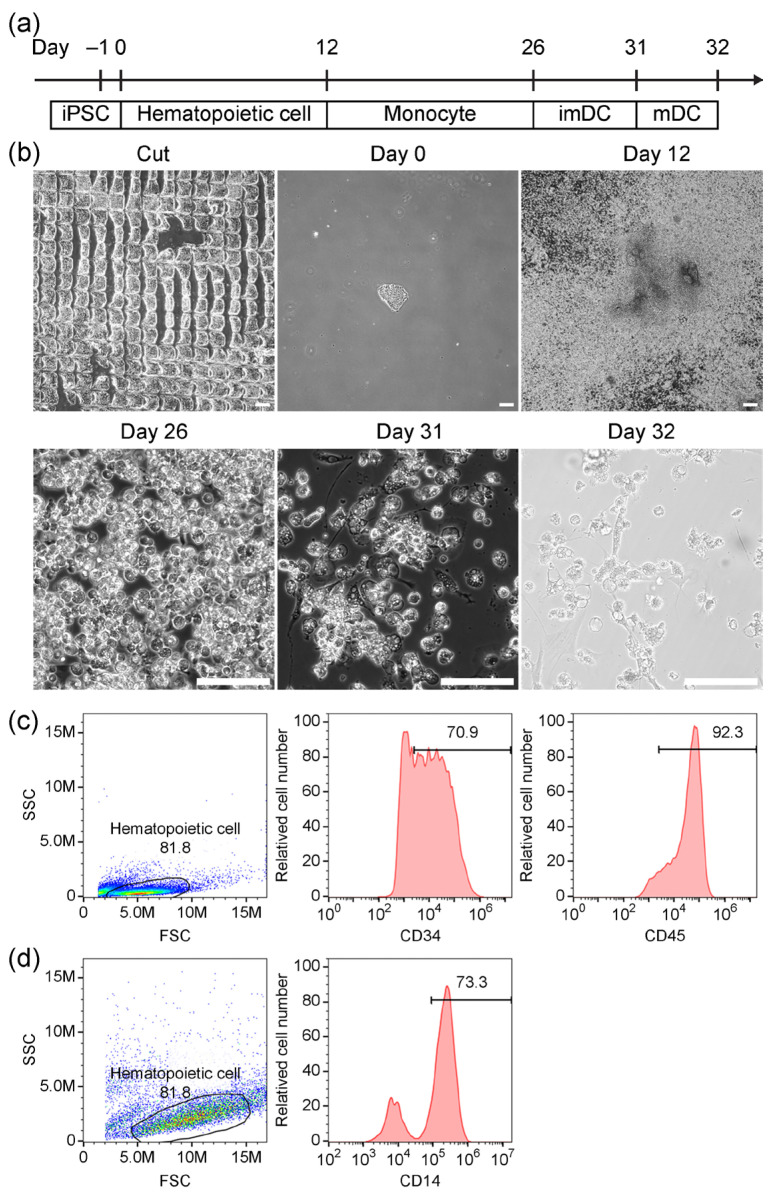
Timeline and intermediate characterization of human-induced pluripotent stem cell (iPSC) differentiation into dendritic cells. (**a**) Timeline of human iPSC differentiation into dendritic cells. (**b**) Representative images of cellular morphology during differentiation at days 0, 12, 26, 31, and 32. Human iPSCs were cut into small pieces before seeding (Scale bar represents 200 μm). (**c**) Characterization of human iPSC-derived hematopoietic stem cells by flow cytometry. CD34 and CD45 were used as surface markers. (**d**) Characterization of human iPSC-derived monocytes by flow cytometry. CD14 was used as the surface marker.

During the iPSC-to-dendritic cell differentiation process, iPSCs were sequentially differentiated into hematopoietic lineage-committed cells, monocytes, and finally dendritic cells, a protocol that required 32 days to complete (Figure 1a). Before initiating differentiation, human iPSCs were cultured for 5–7 days until they reached approximately 80% confluency. The colonies were then mechanically cut into small fragments and gently scraped from the culture surface. After 1 day of incubation, the seeded cell fragments adhered to the culture plate (Figure 1b), indicating the start of the hematopoietic stem cell differentiation process. By day 12, the iPSCs had differentiated into hematopoietic stem cells, with 70.9% of the population expressing CD34 and 92.3% expressing CD45 (Figure 1c). These results confirm the successful induction of hematopoietic lineage commitment from iPSCs.

### 2.2. Differentiation of iPSC-Derived Hematopoietic Cells into Monocytes

Following the generation of hematopoietic stem cells on day 12, monocyte differentiation was initiated by replacing the culture medium with monocyte induction media and continuing the culture for an additional 14 days. By day 26, CD14 expression was detected on 73.3% of the iPSC-derived monocytes (Figure 1b,d). These results confirm the successful differentiation of iPSC-derived hematopoietic cells into functional monocytes.

### 2.3. Phenotypic and Functional Validation of iPSC-Derived Dendritic Cells

iPSC-derived monocytes were purified using anti-CD14 magnetic microbeads. The resulting CD14^+^ monocytes were induced to differentiate into dendritic cells. After 5 days of culture, these cells developed into immature dendritic cells. To promote maturation, various candidate probiotic strains were added and co-cultured for an additional 24 h (Figure 1a). Dendritic-like morphology was observed in the probiotic-activated cells (Figure 1b).

**Figure 2:**
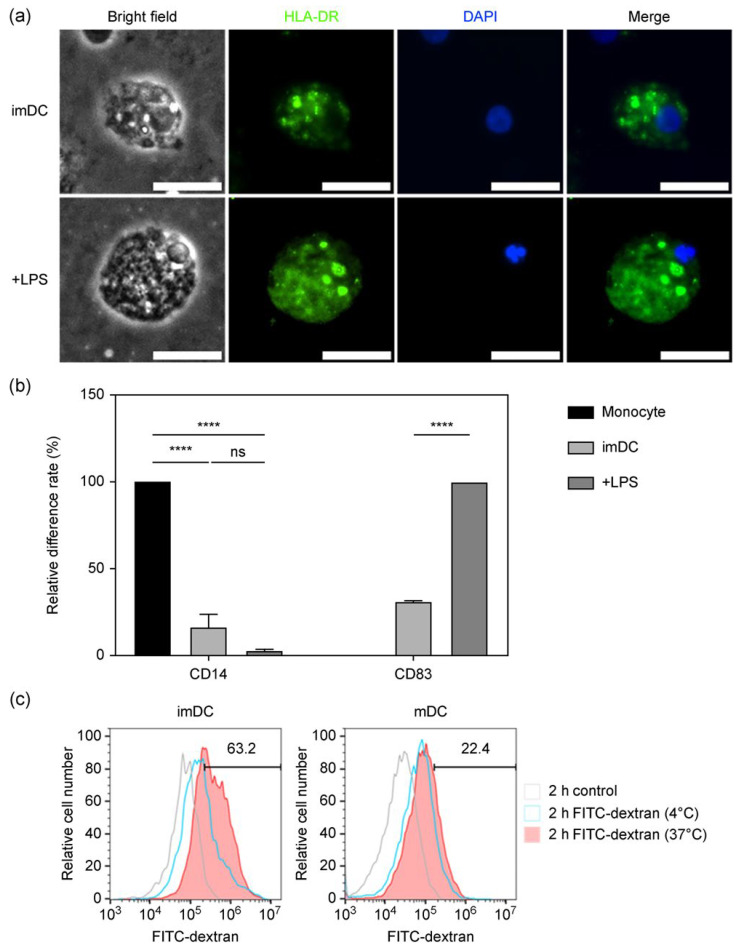
Phenotypic and functional characterization of human iPSC-derived dendritic cells. (**a**) Morphology and immunofluorescence of iPSC-derived dendritic cells with or without lipopolysaccharide (LPS)-induced maturation. Bright-field images show the original cell morphology. HLA-DR is a type of major histocompatibility (MHC) class II molecule that presents antigens to T lymphocytes, and 4′,6-diamidino-2-phenylindole (DAPI) is a fluorescent dye used to stain DNA (Scale bar represents 20 μm). (**b**) Characterization of immature and mature human iPSC-derived dendritic cells by flow cytometry. Monocyte surface marker CD14 and mature dendritic cell surface marker CD83 were used to distinguish the differentiation status between the two cell types (**** *p* < 0.0001, ns: not significant). (**c**) Phagocytosis of fluorescein isothiocyanate (FITC)-dextran by immature and mature human iPSC-derived dendritic cells. In the control group, FITC-dextran was replaced with phosphate-buffered saline (PBS). All samples were incubated for 2 h in the dark. Data shown are representative of at least three independent differentiation batches.

To verify that the iPSC-derived dendritic cells exhibited phenotypic characteristics of primary dendritic cells, immature cells were stained with HLA-DR and 4′,6-diamidino-2-phenylindole (DAPI), with or without lipopolysaccharide (LPS) to induce maturation. HLA-DR expression increased upon LPS stimulation, and the cells were notably larger than unstimulated immature cells (Figure 2a).

Flow cytometry analysis further revealed that, relative to iPSC-derived monocytes, CD14 expression by immature dendritic cells decreased significantly (16.6%) and was further reduced to 2.8% following LPS-induced maturation. Conversely, only 31.4% of immature dendritic cells expressed the maturation marker CD83, which was notably higher in LPS-stimulated mature cells (Figure 2b).

iPSC-derived dendritic cells were incubated with fluorescein isothiocyanate (FITC)-dextran to evaluate their endocytic capacity. After 2 h at 37 °C or 4 °C, iPSC-derived immature dendritic cells exhibited 63.2% uptake of FITC-dextran at 37 °C, whereas LPS-activated dendritic cells showed only 22.4% uptake under the same conditions (Figure 2c). Minimal uptake at 4 °C confirmed that antigen internalization is an active process.

**Figure 3:**
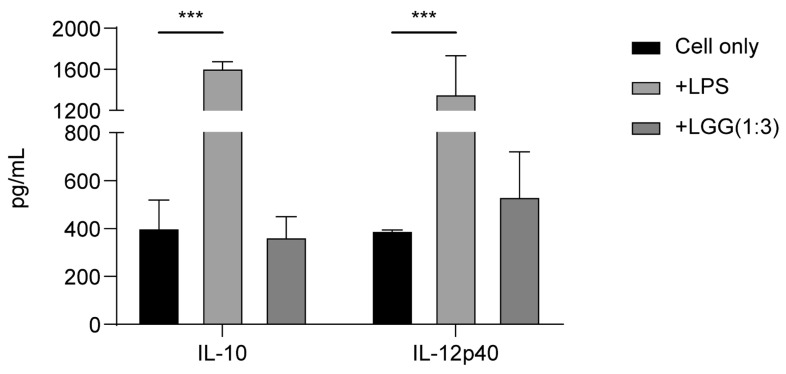
Cytokine secretion assay of human iPSC-derived immature and mature dendritic cells. Interleukin (IL)-10 and IL-12p40 secretion by human iPSC-derived immature dendritic cells after stimulation with LPS or co-culture with *Lactobacillus rhamnosus* GG. The “cell only” group refers to iPSC-derived immature dendritic cells without any stimulation. Plates were incubated for 24 h to measure cytokine secretion into the culture medium (*** *p* < 0.001). Data shown are representative of at least three independent differentiation batches.

The cytokine secretion profile of iPSC-derived dendritic cells was also evaluated under different stimulation conditions: without stimulation, with LPS stimulation, or co-cultured with the well-known probiotic *Lactobacillus rhamnosus* GG. Enzyme-linked immunosorbent assay (ELISA) results confirmed that iPSC-derived dendritic cells secreted cytokines normally in response to LPS stimulation. Furthermore, compared with the cell-only group, co-culture with *L. rhamnosus* GG at a 3:1 bacteria-to-cell ratio resulted in an increase in the production of interleukin (IL)-12p40, whereas IL-10 levels were reduced relative to those in unstimulated cells (Figure 3).

Comparable surface marker expression patterns and cytokine responses were observed across independent differentiation batches, indicating good batch-to-batch reproducibility. These findings validate the use of iPSC-derived dendritic cells as a physiologically relevant and functionally competent model for evaluating immunomodulatory effects in probiotic screening assays.

### 2.4. Cytokine Responses of iPSC-Derived Dendritic Cells Co-Cultured with Lactic Acid Bacteria

**Figure 4:**
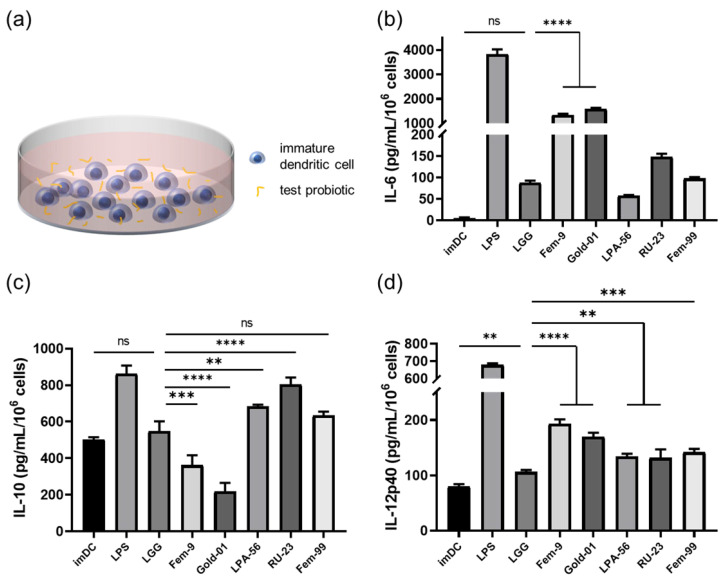
Screening candidate probiotic strains for co-culturing with human iPSC-derived dendritic cells. (**a**) Schematic illustration of the co-culture models of human iPSC-derived dendritic cells and probiotic strains. (**b**) IL-6 secretion by human iPSC-derived dendritic cells after co-culturing with different lactic acid bacteria. *L. rhamnosus* GG was used as the internal control. (**c**) IL-10 secretion by human iPSC-derived dendritic cells after co-culture with different lactic acid bacteria. *L. rhamnosus* GG was used as the internal control. (**d**). IL-12p40 secretion ratio by human iPSC-derived dendritic cells after co-culturing with different lactic acid bacteria. *L. rhamnosus* GG was used as the internal control (** *p* < 0.01, *** *p* < 0.001, **** *p* < 0.0001, ns: not significant). Data shown are representative of at least three independent differentiation batches.

To distinguish the immunomodulatory effects of different lactic acid bacteria on dendritic cells, iPSC-derived immature dendritic cells were co-cultured with different lactic acid bacteria at a 1:3 cell-to-bacteria ratio for 24 h, and the levels of IL-6, IL-10, and IL-12p40 were assessed in the supernatants (Figure 4a).

*L. rhamnosus* GG served as the internal control strain to benchmark cytokine responses among the different bacterial treatments. The cytokine analysis showed that *L. fermentum* Fem-9 and *B. longum* subsp. *longum* Gold-01 induced higher IL-6 secretion from iPSC-derived dendritic cells than *L. rhamnosus* GG, whereas *L. plantarum* LPA-56, *L. reuteri* RU-23, and *L. fermentum* Fem-99 induced lower IL-6 levels (Figure 4b). *L. plantarum* LPA-56 and *L. reuteri* RU-23 stimulated higher IL-10 secretion from iPSC-derived dendritic cells than *L. rhamnosus* GG, whereas *L. fermentum* Fem-9 and *B. longum* subsp. *longum* Gold-01 induced lower IL-10 levels (Figure 4c). All tested probiotics stimulated higher IL-12p40 secretion from iPSC-derived dendritic cells than *L. rhamnosus* GG, with *L. fermentum* Fem-9 and *B. longum* subsp. *longum* Gold-01 exhibiting the highest levels (Figure 4d).

These findings highlight strain-specific differences in dendritic cell cytokine responses and support the utility of iPSC-derived dendritic cells for probiotic screening.

### 2.5. Human CD4^+^ T Cell Cytokine Responses to Probiotic-Activated iPSC-Derived Dendritic Cell Supernatants

**Figure 5:**
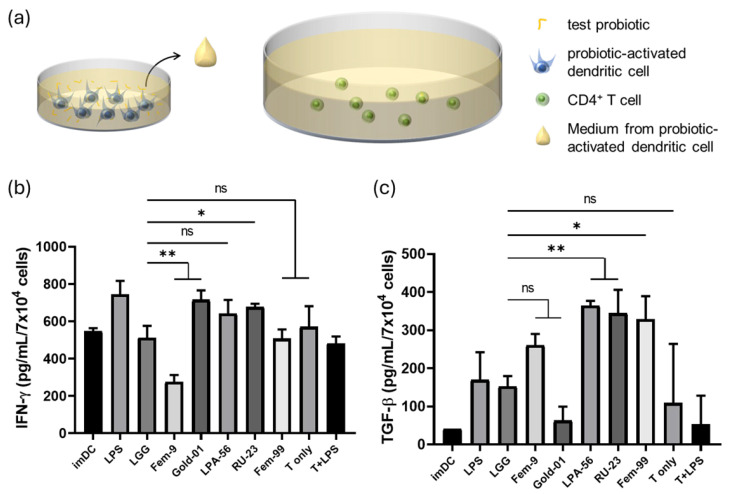
Functional assay of human primary CD4^+^ T cells co-cultured with conditioned medium from human iPSC-derived dendritic cells activated by candidate lactic acid bacteria. (**a**) Schematic illustration of the co-culture model using conditioned medium from human iPSC-derived dendritic cells activated by candidate lactic acid bacteria, applied to human CD4^+^ T cells. (**b**) interferon (IFN)-γ secretion by human CD4^+^ T cells after co-culture with conditioned medium from human iPSC-derived dendritic cells activated by candidate lactic acid bacteria. *L. rhamnosus* GG was used as the internal control. (**c**) Transdermal growth factor (TGF)-β secretion by human CD4^+^ T cells after co-culture with conditioned medium from human iPSC-derived dendritic cells activated by candidate lactic acid bacteria. *L. rhamnosus* GG was used as the internal control (* *p* < 0.05, ** *p* < 0.01, ns: not significant). Data shown are representative of at least three independent differentiation batches.

To further evaluate the ability of probiotic-activated dendritic cells to regulate T cell differentiation, we co-cultured human primary CD4^+^ T cells with supernatants from probiotic-activated iPSC-derived dendritic cells and measured cytokine secretion after 24 h (Figure 5a).

Compared with *L. rhamnosus* GG, *B. longum* subsp. *longum* Gold-01 and *L. reuteri* RU-23 induced higher interferon (IFN)-γ levels, *L. fermentum* Fem-9 induced lower IFN-γ levels, while *L. plantarum* LPA-56 and *L. fermentum* Fem-99 did not induce exert significantly different effects (Figure 5b).

Compared with *L. rhamnosus* GG, *L. plantarum* LPA-56, *L. reuteri* RU-23, and *L. fermentum* Fem-99 induced higher transforming growth factor (TGF)-β secretion, while no significant differences were observed for *L. fermentum* Fem-9 and *B. longum* subsp. *longum* Gold-01 (Figure 5c).

Cytokine levels measured in T cell–only controls were consistently lower and distinct from those induced by conditioned media from probiotic-activated dendritic cells. These differences in IFN-γ and TGF-β production highlight the potential of iPSC-derived dendritic cells as a functional model for screening immunomodulatory effects of probiotics on T cell responses.

## 3. Discussion

In this study, we established a human iPSC-derived dendritic cell platform for evaluating the immunomodulatory effects of lactic acid bacteria. Using this standardized cell-based system, we demonstrated that iPSC-derived dendritic cells exhibit phenotypic and functional characteristics comparable to those of primary dendritic cells, including antigen uptake capacity, cytokine secretion, and responsiveness to the well-characterized probiotic strain *L. rhamnosus* GG. Conventional in vitro evaluation of probiotic immunomodulatory effects commonly relies on dendritic cells differentiated from peripheral blood mononuclear cells or monocytes. Although these models are physiologically relevant, their application is often constrained by donor-to-donor variability, limited cell availability, and batch-to-batch inconsistency. In contrast, iPSC-derived dendritic cells provide a renewable and standardized human cell source that may help mitigate these limitations. In the present study, cytokine response patterns elicited by *L. rhamnosus* GG in iPSC-derived dendritic cells were comparable to those reported for blood-derived dendritic cells, supporting functional comparability between the two systems. Beyond cytokine responsiveness, the iPSC-based platform offers practical advantages in scalability and reproducibility, making it suitable for systematic in vitro screening of immunomodulatory probiotics. Among the assessed strains, *L. plantarum* LPA-56, *L. reuteri* RU-23, and *L. fermentum* Fem-99 were found to induce cytokine profiles conducive to the differentiation of regulatory T (Treg) cells, whereas *B. longum* Gold-01 induced Th1-associated cytokine responses. In contrast, *L. fermentum* Fem-9 appeared to have no clear influence on either Th1 or Treg differentiation. Collectively, these findings validate iPSC-derived dendritic cells as a reproducible and physiologically relevant model for functional probiotic screening.

Upon differentiation into hematopoietic cells, the human iPSC line CPT-01 expresses hematopoietic surface markers CD34 and CD45 at levels of 70.9% and 92.3%, respectively. This differs from the well-known high CD34 expression observed in hematopoietic stem cells [33]; however, it is consistent with the hematopoietic cells directly derived from umbilical cord blood [34,35]. Hence, the iPSC cell line used in this study differentiates into hematopoietic stem cells with outcomes similar to those of cord blood-derived hematopoietic cells.

The CD14 expression levels of monocytes differentiated from iPSC-derived hematopoietic stem cells were also comparable to those of cord blood-derived monocytes. Vuoti et al. compared CD14 expression in monocytes from human peripheral blood, bone marrow, and cord blood, and found that while peripheral blood and bone marrow monocytes exhibited high CD14 expression, cord blood monocytes showed approximately 70% positivity [36]. Stec et al. also reported that cord blood-derived monocytes expressed approximately 60% CD14. They further demonstrated that CD14 expression could be enhanced by adding 1,25-dihydroxyvitamin D_3_ during the differentiation process to increase the expression levels from 62.4% to >80% [37].

The surface marker CD83 is generally recognized as a marker of mature dendritic cells; however, low levels of CD83 can still be detected in immature dendritic cells. Mainali and John reported that immature dendritic cells derived from cord blood expressed low levels of CD83 [38]. Immature dendritic cells derived from other sources also exhibit low CD83 expression. Similarly, Klein et al. reported that CD83 is initially produced intracellularly and subsequently transported to the cell membrane of mature dendritic cells via endocytosis and recycling [39].

Antigen uptake and presentation on the cell surface to activate downstream immune responses is one of the most important functions of dendritic cells. This function is typically associated with their immature stage. Immature dendritic cells possess a strong capacity for antigen uptake, which is significantly reduced upon maturation [40]. Our human iPSC-derived dendritic cells exhibited antigen uptake capacity comparable to that of primary cord blood-derived dendritic cells [41]. This capacity was reduced in LPS-activated mature dendritic cells, consistent with previous findings [42,43].

Several studies have reported that *L. rhamnosus* GG induces dendritic cells to secrete higher levels of IL-12, but not IL-10, both in vitro and in vivo [44,45]. Consistently, our human iPSC-derived dendritic cells exhibited a similar cytokine response after co-culturing with *L. rhamnosus* GG. In addition to morphological and functional assessments, our iPSC-derived dendritic cells exhibited responses similar to those of primary dendritic cells.

Dendritic cells are key antigen-presenting cells that can trigger the differentiation of naïve CD4^+^ T cells into multiple subsets. Each subset of T cells plays a distinct role in the immune system, contributing to the defense against various infections and external stimuli in the human body [46]. A well-known example of immune balance involves the coordinated activities of Th1, Th2, and Treg cells, each contributing to specific aspects of immune regulation.

Cytokines secreted by dendritic cells influence naïve T cell differentiation into distinct subsets. IL-12 is a pro-inflammatory cytokine that promotes the differentiation of naïve T cells into Th1 cells, which contribute to innate resistance and adaptive immunity, helping defend against most bacteria, intracellular protozoa, and fungal pathogens [47]. However, excessive activation of Th1 cells may lead to autoimmune diseases such as type 1 diabetes, rheumatoid arthritis, multiple sclerosis, and Hashimoto’s thyroiditis [48,49]. In contrast, Th2 cells are primarily associated with immune responses against parasites and allergens, and their associated cytokines promote B cell activation [50]. Excessive Th2 cell activation may cause atopic diseases such as asthma, atopic dermatitis, allergic rhinitis, and food allergies [51]. Meanwhile, another group of CD4^+^ T cells, Treg cells, plays a key role in suppressing inflammation, with reported roles in immune tolerance as well as tissue maintenance and repair [52,53].

IL-10 was initially considered a Th2 cell-promoting cytokine. Subsequent studies have shown that IL-10 can inhibit IL-12–driven differentiation of naïve T cells into Th1 cells and is associated with Treg induction and function [54,55]. Bettelli et al. found that excessive IL-6 levels can inhibit Treg cell formation and promote differentiation toward Th17 cells [56]. With the recent growing number of studies on the gut microbiota, probiotics have been reported to enhance IL-10 secretion by co-cultured dendritic cells, thereby promoting anti-inflammatory effects [57,58].

Although the absolute changes in individual cytokine levels were moderate, immune cell fate decisions are governed by the combined cytokine milieu rather than by single cytokines alone. IL-10 plays a critical modulatory role by counterbalancing pro-inflammatory signals such as IL-6 and IL-12, and subtle shifts in this balance can lead to distinct downstream T cell responses. Therefore, evaluating coordinated cytokine patterns relative to a well-established reference strain provides a biologically meaningful framework for functional probiotic screening.

Using our iPSC-derived dendritic cell platform, we evaluated for the immunomodulatory effects of five lactic acid bacterial strains, among which, *L. plantarum* LPA-56, *L. reuteri* RU-23, and *L. fermentum* Fem-99 stimulated the dendritic cells to secrete high levels of IL-10 and low levels of IL-6. When co-cultured with human CD4^+^ T cells, these dendritic cells markedly increased TGF-β and slightly raised IFN-γ levels compared with *L. rhamnosus* GG, suggesting that the dendritic cells supported Treg differentiation. Similar IL-10–inducing effects have been observed for *L. plantarum* OLL2712, which enhances anti-inflammatory cytokine production in dendritic cells [59]. *B. longum* subsp. *longum* Gold-01–stimulated iPSC-derived dendritic cells secreted high levels of IL-12p40 and IL-6, but low IL-10. Co-cultured CD4^+^ T cells subsequently produced high IFN-γ and low TGF-β, indicating that the dendritic cells promoted Th1 cell differentiation, consistent with previous findings that certain *B. longum* strains promote Th1-type immune responses [60]. *L. fermentum* Fem-9–stimulated iPSC-derived dendritic cells produced high levels of IL-12p40 and IL-6, but low IL-10. The co-cultured CD4^+^ T cells secreted lower IFN-γ and non-statistically significant levels of TGF-β compared with *L. rhamnosus* GG. These results suggest that, compared with *B. longum* subsp. *longum* Gold-01, *L. fermentum* Fem-9 induced a relatively higher IL-10 response, which may have limited the extent of Th1 cell differentiation in the co-cultured CD4^+^ T cells.

Collectively, these findings highlight the potential applicability of iPSC-derived dendritic cells as a model for the functional evaluation of probiotic strains that eliminates the need for blood-derived dendritic cells and minimizes dependence on the use of animals in immunological assays. By reproducing key phenotypic and cytokine responses characteristic of primary dendritic cells, this system provides a human-relevant platform for investigating how bacterial stimulation modulates dendritic cell functions and influences T cell polarization. Several limitations of this study should be acknowledged. Functional screening was performed using a limited number of lactic acid bacterial strains and a single human iPSC line (CPT-01), which may restrict the generalizability of the present findings and supports the reliability of the proposed platform within the defined experimental scope. In addition, total CD4^+^ T cells were used in the co-culture assays; however, the inclusion of T cell–only controls allowed reliable discrimination of dendritic cell–driven immunomodulatory effects. Nevertheless, a key advantage of the iPSC-derived dendritic cell system lies in its scalability and flexibility. The use of iPSCs enables the generation of standardized dendritic cells from donors of different genetic backgrounds, ages, sexes, or disease conditions. Future studies incorporating multiple iPSC lines, naïve CD4^+^ T cells, and a broader spectrum of probiotic strains will facilitate systematic and precision-oriented evaluation of immunomodulatory probiotics in diverse immune contexts.

## 4. Materials and Methods

### 4.1. Human iPSC Culture

The human iPSC line CPT-01 was obtained from the Center for Regenerative Medicine and Cellular Therapy at National Yang Ming Chiao Tung University. Before use, surface markers of pluripotency (SSEA4 and TRA-1–60) and stem cell-associated transcription factors (OCT4 and SOX2) were confirmed by flow cytometry. Approximately 1.2 × 10^5^ iPSCs were cultured in StemFlex™ medium (Gibco™, Waltham, MA, USA, #A3349401), supplemented with 10 nM/mL Y-27632 (STEMCELL Technologies, Vancouver, BC, Canada, #72304) and iMatrix-511 silk (#892021), for the first 24 h in a 6 cm dish (GeneDireX, Taoyuan, Taiwan, #PC203-0600) under 5% CO_2_ at 37 °C. The medium was refreshed daily for 1 week until the cells reached nearly 70–80% confluence and were passaged. Cells were washed once with Dulbecco’s Phosphate-Buffered Saline (DPBS; Gibco™, Waltham, MA, USA, #14190250) and detached with Accutase^®^ (Innovative Cell Technologies, Inc., San Diego, CA. USA, #AT104) for 2 min at 37 °C. Accutase was diluted with DPBS and removed by centrifugation at 1000 rpm for 3 min. Cells were then seeded for maintenance culture. This study did not involve the derivation of new human induced pluripotent stem cells. The human iPSC line used was previously established and provided by the Center for Regenerative Medicine and Cellular Therapy, National Yang Ming Chiao Tung University. Therefore, institutional review board approval was not required.

### 4.2. Differentiation of Human iPSCs into Hematopoietic Cells and Monocytes

Hematopoietic cells were differentiated from iPSCs using the STEMdiff™ Hematopoietic Kit (STEMCELL Technologies, Vancouver, BC, Canada, #05310), with modifications to the seeding process [61]. Briefly, iPSCs at nearly 70–80% confluence were washed once with StemFlex medium and placed in a fresh medium. The cells were cut into small pieces using a Passaging Tool (Gibco™, Waltham, MA, USA, #23181010), and the fragments were gently scraped and counted. Approximately 100 undifferentiated iPSC pieces were seeded per well in a laminin-coated 6-well plate and incubated for 1 day. The following day, the plates were examined under a light microscope to confirm that approximately 40–100 iPSC colonies had adhered within each well of the six-well plate, in accordance with the manufacturer’s recommendations. Mature hematopoietic cells were obtained on day 12, and their surface markers were analyzed by flow cytometry.

Monocytes were differentiated from mature hematopoietic cells using SFEM II medium (STEMCELL Technologies, Vancouver, BC, Canada, #09605) supplemented with Monocyte Differentiation Supplement (STEMCELL Technologies, Vancouver, BC, Canada, #05324), in accordance with the manufacturer’s recommendations [62]. By day 14, mature monocytes had developed and were analyzed for surface markers by flow cytometry.

### 4.3. CD14-Positive Cell Selection

At the end of monocyte differentiation on day 14, the cells were collected and passed through a 40 μm cell strainer (GeneDireX, Taoyuan, Taiwan, #PC728-0100). CD14^+^ monocytes were enriched using the Human CD14 Positive Selection Kit II (STEMCELL Technologies, Vancouver, BC, Canada, #17858) and adjusted to a final density of 1 × 10^6^ cells/mL before downstream functional assays or differentiation was performed.

### 4.4. Differentiation and Maturation of Human iPSC-Derived Dendritic Cells

Immature dendritic cells were generated from human iPSC-derived monocytes using the ImmunoCult™ Dendritic Cell Culture Kit (STEMCELL Technologies, Vancouver, BC, Canada, #10985) [63]. Briefly, CD14^+^ monocytes were adjusted to a density of 1 × 10^6^ cells/mL and seeded into 24-well plates. Fresh complete medium was replaced on day 3, and immature dendritic cells were obtained on day 5. The cells were subsequently used for co-culture experiments with various probiotics for 24 h, with LPS (0.5 μg/mL, Sigma, St. Louis, MO, USA, #L4516) serving as a positive control.

### 4.5. FITC-Dextran Uptake Assay

For the in vitro dendritic cell uptake assay, immature dendritic cells and LPS-treated mature dendritic cells were harvested and incubated with 1 mg/mL FITC-dextran (Sigma, #FD40S) for 2 h at either 37 °C or 4 °C. Phagocytic activity was assessed by flow cytometry, and data were analyzed using FlowJo software (v10.8.1, BD Biosciences, Ashland, OR, USA).

### 4.6. Bacterial Culture and Preparation

Five lactic acid bacterial strains, *Lactiplantibacillus plantarum* LPA-56, *Limosilactobacillus reuteri* RU-23, *Lactobacillus fermentum* Fem9, *Limosilactobacillus fermentum* Fem-99, and *Bifidobacterium longum* subsp. *longum* Gold-01, were kindly provided by PbMed Biotech Co., Ltd. (Tainan, Taiwan). Taxonomic identification at the genus and species levels was performed by the supplier prior to this study. *Lactobacillus rhamnosus* GG (ATCC 7469) was kindly provided by Bio-Race Biotech Co., Ltd. (Hangzhou, China). *L. plantarum* LPA-56, *L. reuteri* RU-23, *L. fermentum* Fem9, *L. fermentum* Fem-99, and *L. rhamnosus* GG were cultured in de Man, Rogosa, and Sharpe (MRS) broth (BD Biosciences, Ashland, OR, USA, #288130) at 37 °C under anaerobic conditions for 2–3 days. *B. longum* subsp. *longum* Gold-01 was cultured in MRS broth supplemented with 0.05% cysteine at 37 °C under anaerobic conditions for 2–3 days.

All bacterial strains were adjusted to 1 × 10^8^ CFU/mL, washed twice with PBS, centrifuged at 10,000× *g* for 10 min, and then frozen in 1 mL aliquots of PBS containing 50% glycerol at −80 °C until use.

### 4.7. Stimulation of Human iPSC-Derived Dendritic Cells with Lactic Acid Bacteria

Immature dendritic cells (1 × 10^6^ cells/mL) were stimulated with live frozen lactic acid bacteria, with viable bacterial counts determined post-thaw, at a defined 1:3 ratio of iPSC-derived dendritic cells to viable bacterial CFU under 5% CO_2_ at 37 °C for 24 h. Prior to stimulation, frozen bacteria were thawed, washed twice with PBS, centrifuged at 10,000× *g* for 10 min, and resuspended in dendritic cell medium. To account for potential loss of bacterial viability during frozen storage, all bacterial strains were subjected to post-thaw CFU enumeration prior to each co-culture experiment. Briefly, bacteria were thawed, serially diluted, plated on MRS agar, and incubated under anaerobic conditions to determine viable CFU. Based on these counts, bacterial suspensions were adjusted to achieve the desired cell-to-bacteria ratio. LPS was used as a positive control at 0.5 μg/mL. The culture supernatants were collected and analyzed for IL-10, IL-12p40, and IL-6 using ELISA kits (R&D Systems, Minnneapolis, MN, USA) according to the manufacturer’s instructions.

### 4.8. Human CD4^+^ T Cell Expansion and Stimulation

Human primary CD4^+^ T cells were commercially obtained from an overseas supplier as de-identified research materials (STEMCELL Technologies, Vancouver, BC, Canada). Purified human peripheral blood CD4^+^ T cells were resuspended in Roswell Park Memorial Institute 1640 supplemented with 10% heat-inactivated fetal bovine serum and 50 μM β-mercaptoethanol at a final concentration of 1 × 10^6^ cells/mL. Dynabeads™ Human T-Activator CD3/CD28 (Gibco™, Waltham, MA, USA, #11131D) were added to the T cell culture at a 1:1 bead-to-cell ratio after a 16 h recovery period. The cells were cultured under 5% CO_2_ at 37 °C for 3 days.

For the T cell co-culture experiment, cell-free conditioned medium from probiotic-stimulated dendritic cells was collected and centrifuged at 10,000 rpm for 10 min. Viable CD4^+^ T cells were enriched using the EasySep™ Dead Cell Removal (Annexin V) Kit (STEMCELL Technologies, Vancouver, BC, Canada, #17899) and re-stimulated with Dynabeads™ Human T-Activator CD3/CD28 at a 1:1 bead-to-cell ratio. The viable CD4^+^ T cells were seeded at 1 × 10^6^ cells/mL in 96-well plates and treated with a three-fold diluted, cell-free medium from dendritic cells which lactic acid bacteria activated one day, under 5% CO_2_ at 37 °C for 24 h. The culture medium were collected by centrifuged at 1500× *g* for 10 min to obtain the first supernatant to remove T cells, then centrifuged at 10,000× *g* for 10 min to collect cell-free supernatants. IFN-γ and TGF-β were analyzed using ELISA kits (R&D Systems) according to the manufacturer’s instructions.

Due to limited availability of purified naïve CD4^+^ T cells, total peripheral CD4^+^ T cells were used in this study. A T cell–only control was included in all experiments to account for baseline cytokine secretion. This study did not involve the collection of human samples. The primary human CD4^+^ T cells were commercially obtained as de-identified research materials. Therefore, institutional review board approval was not required.

### 4.9. Flow Cytometry

The cells were harvested and resuspended in DPBS. Each sample was adjusted to 1 × 10^6^ cells, washed with DPBS, centrifuged at 1000 rpm for 5 min, and stained with fluorophore-conjugated primary antibodies for 1 h at 4 °C. After being washed three times with DPBS, the cells were then analyzed using an Accuri™ C6 flow cytometer (BD Biosciences, Ashland, OR, USA), and data were analyzed using FlowJo software. The antibodies used included: CD34-PE (Proteintech, Rosemont, IL, USA, #PE-65183), CD45-PE (BD Biosciences, Ashland, OR, USA, #555483), CD14-FITC (BD Biosciences, Ashland, OR, USA, #555397), CD11c-FITC (Proteintech, Rosemont, IL, USA, #FITC-65086), and CD83-PE (BD Biosciences, Ashland, OR, USA, #550634).

### 4.10. Immunofluorescent Staining

The iPSC-derived dendritic cells were centrifuged at 1200 rpm for 10 min, and the supernatant was discarded. The cells were fixed in 4% paraformaldehyde for 15 min, permeabilized with 0.5% Triton X-100 in PBS for 10 min, and then blocked with 1% bovine serum albumin (BSA) in PBS for 1 h at 25 °C. The samples were incubated overnight at 4 °C with the primary antibody against HLA-DR (1:100; mouse anti-human HLA-DR; Merck, Darmstadt, Germany, #SAB4700731) diluted in 1% BSA, followed by incubation with the secondary antibody (1:1000; Alexa Fluor 488 goat anti-mouse; Invitrogen, Carlsbad, CA, USA, #A-11001) at 25 °C for 1 h in the dark. All samples were counterstained with DAPI (Invitrogen, Carlsbad, CA, USA, #R37606) for nuclear staining, washed with PBS, and mounted on glass slides using ProLong™ Diamond Antifade Mountant (Invitrogen, Carlsbad, CA, USA, #P36965).

Images were captured using a fluorescence microscope (Olympus Corporation, Hachioji, Japan) and visualized with VisiView^®^ software v4.5.0.7. (Visitron Systems GmbH, Puchheim, Germany).

### 4.11. Cytokine Quantification by ELISA

Culture supernatants from mature dendritic cells stimulated with lactic acid bacteria or LPS, as well as from immature dendritic cells, were collected after 24 h of co-culture and centrifuged at 1000 rpm for 5 min. Supernatants from the bacteria-stimulated groups were further centrifuged at 10,000× *g* for 10 min to remove residual bacteria. All samples were stored at −80 °C until use. The culture supernatants were analyzed using commercially available ELISA kits to measure IL-6 (R&D, #DY206-05), IL-10 (R&D Systems, #DY217B-05), and IL-12p40 (R&D Systems, #DY1240-05), following the manufacturer’s instructions. For the co-culture of probiotic-activated dendritic cell conditioned medium with human CD4^+^ T cells, the supernatants were harvested after 24 h of incubation and centrifuged at 1000 rpm for 5 min. These supernatants were then analyzed using commercially available ELISA kits to quantify IFN-γ (R&D Systems, #DY285B-05) and TGF-β (R&D Systems, #DY240-05), following the manufacturer’s instructions. Cytokine concentrations were quantified using standard curves generated from serial dilutions of recombinant cytokines provided with each ELISA kit. Results are presented as the mean ± standard error of the mean (SEM) in pg/mL from three independent experiments.

### 4.12. Statistical Analysis

All experiments, including iPSC-to-dendritic cell differentiation and subsequent co-culture assays, were independently performed using at least three complete batches (*n* ≥ 3), each representative an independent biological replicate. Data are presented as mean ± SEM. ELISA data were analyzed using a four-parameter logistic curve fitting model. Statistical comparisons were performed using one-way analysis of variance followed by Dunnett’s multiple comparisons test in GraphPad Prism software (v8.3, Boston, MA, USA). *p*-values < 0.05 were considered statistically significant.

## 5. Conclusions

In this study, we succeeded in establishing a human iPSC-derived dendritic cell platform that can be applied in in vitro evaluations of the immunomodulatory effects of lactic acid bacteria. These iPSC-derived dendritic cells were found to have phenotypic and functional features comparable to those of primary dendritic cells and showed differential responses to bacterial stimulation.

In the case of *L. plantarum* LPA-56, *L. reuteri* RU-23, and *L. fermentum* Fem-99, conditioned medium derived from probiotic-activated dendritic cells was found to have immunomodulatory effects when applied to human CD4^+^ T cells, promoting cytokine profiles consistent with regulatory T (Treg) differentiation, whereas *B. longum* Gold-01 elicited Th1-associated responses. These findings accordingly indicate that iPSC-derived dendritic cells can provide a reproducible and physiologically relevant human in vitro platform for identifying immunoregulatory bacterial candidates.

## Data Availability

The original contributions presented in this study are included in the article. Further inquiries can be directed to the corresponding authors.

## Abbreviations

The following abbreviations are used in this manuscript:

iPSCs	Induced pluripotent stem cells
DAPI	4′,6-diamidino-2-phenylindole
LPS	Lipopolysaccharide
FITC	Fluorescein isothiocyanate
IL	Interleukin
TGF	Transdermal growth factor
ELISA	Enzyme-linked immunosorbent assay
IFN	Interferon
PBS	Phosphate-buffered saline
DPBS	Dulbecco’s phosphate-buffered saline
BSA	Bovine serum albumin
Tregs	Regulatory T cells
